# Bioinformatic Analysis Identifies Potential Key Genes in the Pathogenesis of Melanoma

**DOI:** 10.3389/fonc.2020.581985

**Published:** 2020-10-16

**Authors:** Yanjie Han, Xinxin Li, Jiliang Yan, Chunyan Ma, Xin Wang, Hong Pan, Xiaoli Zheng, Zhen Zhang, Biao Gao, Xin-Ying Ji

**Affiliations:** ^1^Clinical Laboratory, Functional Laboratory and Department of Stomatology, Kaifeng Central Hospital, Kaifeng, China; ^2^Hospital Infection Control Office, First Affiliated Hospital of Henan University, Kaifeng, China; ^3^Kaifeng Key Laboratory for Infectious Diseases and Biosafety, Henan International Joint Laboratory of Nuclear Protein Regulation, Henan School of Basic Medical Sciences, Henan University College of Medicine, Kaifeng, China

**Keywords:** melanoma, differentially expressed genes, bioinformatics analysis, hub genes, tumor marker

## Abstract

Melanoma is the deadliest skin tumor and is prone to distant metastases. The incidence of melanoma has increased rapidly in the past few decades, and current trends indicate that this growth is continuing. This study was aimed to explore the molecular mechanisms of melanoma pathogenesis and discover underlying pathways and genes associated with melanoma. We used high-throughput expression data to study differential expression profiles of related genes in melanoma. The differentially expressed genes (DEGs) of melanoma in GSE15605, GSE46517, GSE7553, and the Cancer Genome Atlas (TCGA) datasets were analyzed. Differentially expressed genes (DEGs) were identified by paired t-test. Then the DEGs were performed cluster and principal component analyses and protein–protein interaction (PPI) network construction. After that, we analyzed the differential genes through bioinformatics and got hub genes. Finally, the expression of hub genes was confirmed in the TCGA databases and collected patient tissue samples. Total 144 up-regulated DEGs and 16 down-regulated DEGs were identified. A total of 17 gene ontology analysis (GO) terms and 11 pathways were closely related to melanoma. Pathway of pathways in cancer was enriched in 8 DEGs, such as junction plakoglobin (JUP) and epidermal growth factor receptor (EGFR). In the PPI networks, 9 hub genes were obtained, such as loricrin (LOR), filaggrin (FLG), keratin 5 (KRT5), corneodesmosin (CDSN), desmoglein 1 (DSG1), desmoglein 3 (DSG3), keratin 1 (KRT1), involucrin (IVL), and EGFR. The pathway of pathways in cancer and its enriched DEGs may play important roles in the process of melanoma. The hub genes of DEGs may become promising melanoma candidate genes. Five key genes FLG, DSG1, DSG3, IVL, and EGFR were identified in the TCGA database and melanoma tissues. The results suggested that FLG, DSG1, DSG3, IVL, and EGFR might play important roles and potentially be valuable in the prognosis and treatment of melanoma. These hub genes might well have clinical significance as diagnostic markers.

## Introduction

Melanoma is the most lethal tumor of skin tumors, and prone to distant metastasis (1, 2). The incidence of melanoma has increased rapidly over the past few decades, and current trends indicate that this growth has still been continuing (3–5). Despite encouraging trends related to improved screening and the introduction of new therapies, melanoma remains a major public health problem (6, 7). In 2020, there were approximately 100,350 newly diagnosed melanomas and 6850 deaths worldwide (8).

There are currently an estimated 1.2 million melanoma survivors in the United States alone (9). While many previous studies have examined factors associated with survival (10–13), exhaustive research on the pathogenic genes and markers of melanoma pathogenicity remain scarcely. Data on tumor markers of melanoma can generate important information that can guide treatment, monitoring plans, and point the way for future melanoma research.

In recent years, microarrays and high-throughput sequencing technologies that detect the expression levels of tens of millions of genes in humans have been widely used to predict potential targets for melanoma treatment (14, 15).

Most current studies only focus on the results of a single genetic event or a single cohort study of melanoma (16–18). However, there is still no comprehensive multi-factor analysis and treatment method (19–21). In this study, we have compiled the GEO and TCGA databases in order to explore the key genes and prognostic indicators of melanoma as comprehensively as possible. Our results will promote our cognition of the genetic etiology of melanoma and provide new insights into the clinical diagnosis and treatment of melanoma.

## Materials and Methods

### Data Searches

We conducted a search of Gene Expression Omnibus (GEO: https://www.ncbi.nlm.nih.gov/geo/) for high-throughput functional genomics experiments of melanoma. GSE15605, GSE46517, and GSE7553 themicroarray expression profiling datasets were downloaded from Gene Expression Omnibus. These datasets were based on GPL570, GPL96, GPL570 Affymetrix Human Genome Array Platform, respectively. We used the following search terms: melanoma, primary melanoma, metastasis melanoma and skin cutaneous melanoma. Datasets were screened for dataset record following the criteria: (1) samples contained melanoma and normal skin tissue, (2) study type was restricted to expression profiling by array, (3) organism was restricted to Homo sapiens, (4) original data were accessible. We excluded studies of less than five samples in each group. The gene expression profiles meeting inclusion criteria were selected from GEO database and TCGA (https://cancergenome.nih.gov/abouttcga/overview) database. In addition, RNASeqV2 data for cutaneous melanoma can be downloaded from the TCGA database. Both GEO and TCGA have significantly increased our understanding of cancer. One very evident advantage of GEO and TCGA is that data from different independent studies can be integrated to get a large number of clinical samples based on TCGA HNSC RNA-seq data and indicated good performance for predicting 5-year overall survival. This powerful prognostic marker was successfully verified in another independent patients cohort.

### Data Pre-Processing and Differential Expression Analysis

Robust multi-array average (RMA) approach was performed for background correction and normalization. Then, the original GEO data were converted into expression measures using Affy R package. Limma R package was subsequently employed for identifying DEGs. For TCGA data, edgeR package was used for DEGs screening. Adjusted p < 0.05 and (∣logFC fold change∣) >2 were chosen as the cut-off criteria based on Benjamini & Hochberg (BH) procedure (22). Intersect function in R was applied for identifying the common DEGs among GSE15605, GSE46517 and GSE7553 and TCGA. The Venn diagram was generated by Venn Diagram R package.

### Gene Ontology and Pathway Enrichment Analyses

Gene ontology analysis (GO, http://www.geneontology.org/) was used to identify characteristic biological attributes for DEGs (23). Kyoto Encyclopedia of Genes and Genomes pathway (KEGG) enrichment analysis was performed to identify functional attributes for DEGs (24). We used online database for annotation, visualization and integrated discovery (DAVID) (25) and KEGG to access GO and pathway enrichment analysis. p < 0.05 was set as the cut-off criterion.

### PPI Network Construction

Functional interactions between proteins can provide support for elucidating the molecular mechanisms of disease processes. In our study, the search tool for the retrieval of interacting gene (STRING) (26) database was utilized to construct PPI network. In addition, Cytoscape software was applied to construct protein interaction relationship network (27).

### Hub Genes Identification

The Cytoscape was performed to scale degree and closeness of the PPI network. The degree of a node is the average number of edges (interactions) incident to this node. The genes at the top of the degree distribution (≥ 95% percentile) in the significantly perturbed networks were defined as hub genes.

### Module Analysis of the PPI Network

Module analysis of the PPI network was performed with the parameters of minimum size > 3 and P-Value < 0.01 using ClusterONE, a Cytoscape plugin that unified different clustering techniques and displayed them in a single interface.

### Hub Genes Expression Level and Survival Analysis

Gene-level correlations with patient survival were featured in UALCAN (http://ualcan.path.uab.edu/analysis.html) (28). Available TCGA patient survival data were used for Kaplan–Meier survival analysis and to generate overall survival plots.

### Immunohistochemistry

All clinical specimens used in this research were from the First Affiliated Hospital of Henan University, including 63 cases of melanoma tissues and matched adjacent normal skin tissues obtained from patients who underwent surgery for melanoma or other non-melanoma diseases. Histological diagnosis and tumor stage were determined with immunohistochemical method according to the 2004 World Health Organization guidelines for classification. Paraffin sections (4 μm) were stained overnight with rabbit anti-LOR, anti-FLG, anti-KRT5, anti-CDSN, anti-DSG1, anti-DSG3, anti-KRT1, anti-IVL and anti-EGFR antibody (1: 50; Abcam, UK) at 4°C. Secondary staining was then performed with HRP-conjugated anti-rabbit or anti-mouse IgG using the MaxVision kit and DAB Peroxidase Substrate Kit (Maixin, China). Finally, all slides were stained with hematoxylin. The irrelevant rabbit IgG was used as a control for the primary antibody. All of these were evaluated by assessing staining intensity and percentage of positive cells as follows: no staining (0), weak staining (1), moderate staining (2), and strong staining (3); percentage of positive cells <1% (0), 1–33% (1), 34–66% (2), and 67–100% (3). The two scores for each slide were then combined to produce a final grade of the protein of target gene expression(PTGE): 0, total score = 0; 1+, total score = 1–2; 2+, total score = 3–4; 3+, total score = 5–6. The average score is used when there exists a difference between the two pathologists. This study was approved by the local ethics committee and written informed consent was obtained from each patient.

## Results

### Search Results and Study Characteristics

**Figure 1:**
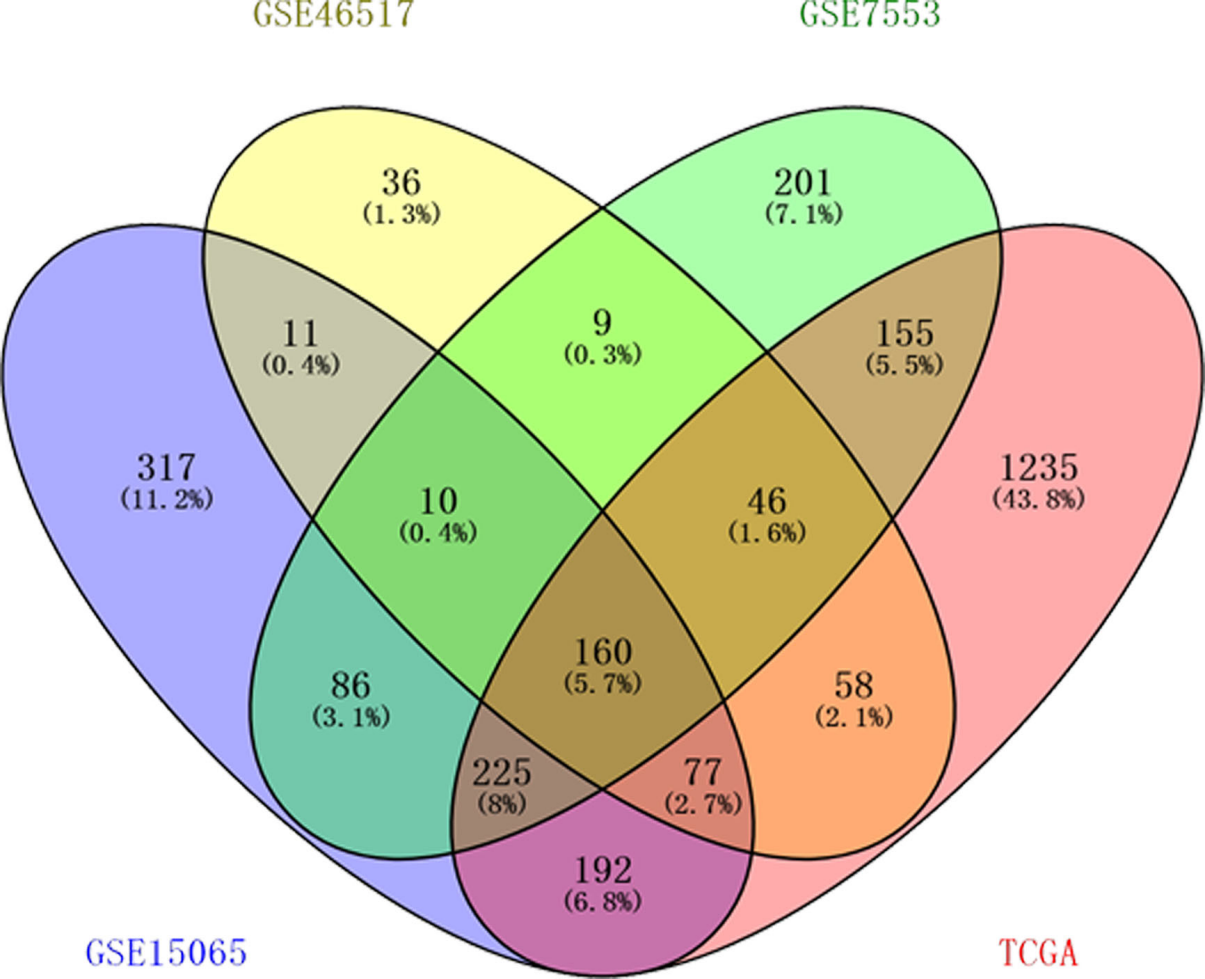
One hundred sixty differentially expressed genes (DEGs) were identified from four datasets. Among them, 144 genes were up-regulated while 16 genes were down-regulated. Different color areas represented different datasets. The cross areas meant the commonly changed DEGs.

According to the inclusion criteria, three GEO datasets and TCGA dataset were obtained in our study: GSE15605, GSE46517, GSE7553, and TCGA skin cutaneous melanoma data. DEGs 1,078, 407, 892, and 2,148 from the expression profile datasets GSE15605, GSE46517, GSE7553, and TCGA dataset were extracted, respectively. 160 consistently expressed genes were identified by Venn analysis (**Figure 1**). Among them, 144 genes were up-regulated while 16 were down-regulated compared to normal skin tissue (**Table 1**).

**Table 1:** 160 differentially expressed genes (DEGs) were identified from datasets.

DEGs	
**Up-regulated genes**	LOR KRT15 GATA3 FLG PKP1 LGALS7B LGALS7 FGFR3 POF1B EFNA3 CST6 KRT2 CDSN COL17A1 DSG1 TRIM29 LY6D SCEL SERPINB5 TACSTD2 LCE2B PPL DSC1 SFN RAPGEFL1 CRCT1 KLK11 KRT23 CCL27 IL37 CA12 C1orf116 KRT1 KLF5 CALML5 PTK6 KRT5 DSC3 HOPX CLCA2 EVPL CHP2 FGFR2 JUP CWH43 ZNF750 POU2F3 ELOVL4 LYPD3 S100A14 KLK5 ANK3 EPHB6 SERPINB7 AIM1L RORA EGFR CXCL14 ANXA8 ANXA8L1 BBOX1 SCNN1A GJB5 ALDH3B2 CXADR PSORS1C2 ADIRF CYP4B1 PERP KLK10 AQP3 KLK7 HAL ASS1 SDC1 EPHX3 TP63 AZGP1 CYP3A5 RAB25 CALML3 KCNK7 EXPH5 TACC2 SPINK5 NEBL DSG3 ADH1B FERMT1 PKP3 DUOX1 CEBPA SLC24A3 ALOX15B CDS1 PTPRF HLA-DQB2 ARHGEF4 GJB3 PDZD2 CLEC3B NMU SPINT2 BCL11A LY6G6C MMP28 C1orf68 SCGB1D2 GPR87 F2RL1 DEFB1 LAD1 LAMB4 MAOA ALOXE3 PAMR1 NTRK2 SLC15A1 ARG1 KRT19 HLF C1orf106 PDZK1IP1 PALMD RNF39 PPP1R13L AIM1 ACSBG1 AKR1C2 PLLP NPY1R AP1M2 KRT31 KRT7 FAT2 PTGS1 IVL CDHR1 ZBTB16 SCNN1B IRF6 ATP2C2 IRX4 ACSL1
**Down-regulated genes**	TRIB2 SLC16A4 SNX10 BCL2A1 AP1S2 ALX1 UPP1 PHLDA1 SOX10 ETV5 PLAT LEF1 CITED1 IGF2BP3 SERPINE2 SPP1

Among them, 144 genes were up-regulated and 16 genes were down-regulated.

### Gene Ontology Analysis

**Figure 2:**
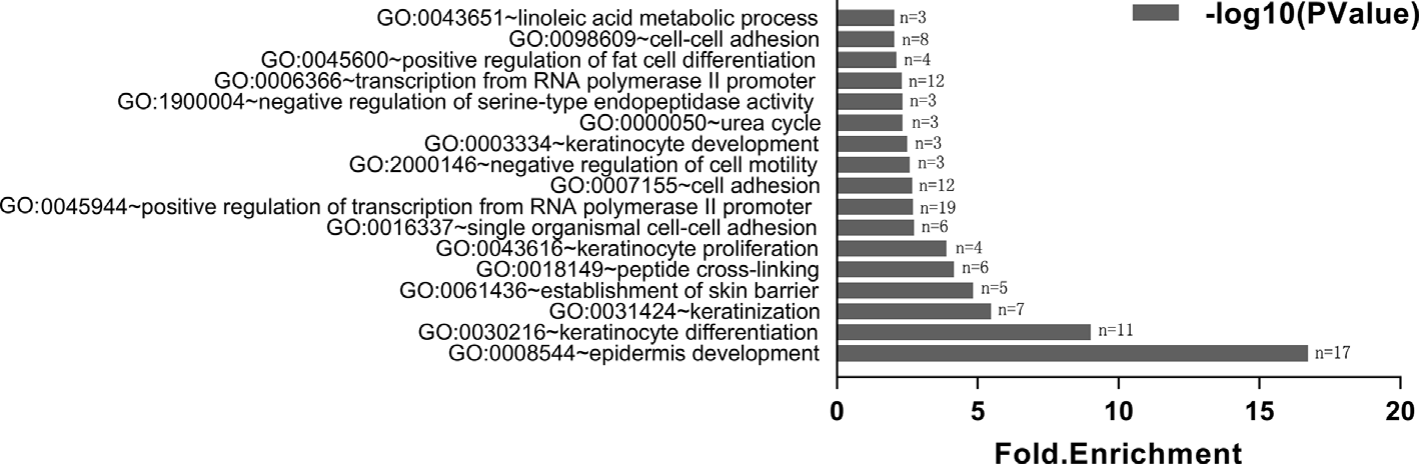
Gene ontology analysis of DEGs. X-axis reflects gene count (n); Y-axis reflects different GO terms. The column value reflects p value (− log10(P Value)): the highest bar represents the biggest − log10(P Value) value (*p < 0.01). We only show terms with p-values less than 0.01 in the figure.

Gene ontology describes gene function and relationships between these concepts. P < 0.01 was used as the cut-off criterion. DEGs were classified into the biological process: pathways and larger processes made up of the activities of multiple gene products. After GO enrichment analysis, we found that 160 DEGs were enriched in 17 GO terms (biological process). Among them, the most enriched GO terms were epidermis development (p=1.88E-17), keratinocyte differentiation (p = 9.90E-10), keratinization (p = 3.40E-06) and establishment of skin barrier (p = 1.49E-05) (**Figure 2**).

### Pathway Enrichment Analysis

**Figure 3:**
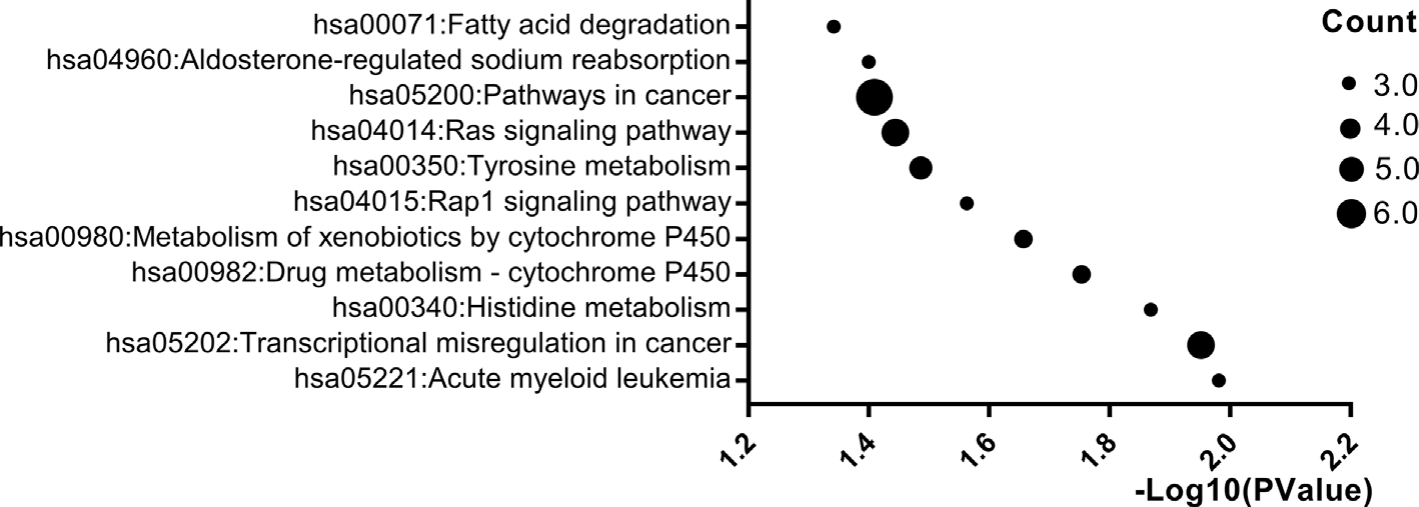
Significantly enriched pathway terms of DEGs. X-axis reflects p value [− log10(p value)]. Y-axis reflects different passway terms. The node size reflects gene count: the bigger the gene count, the bigger the node size is (*p < 0.05). We only showed items with p-values less than 0.05 in the figure.

Pathway enrichment analysis was carried out by online websites of KEGG, a database was applied to assign sets of DEGs to specific pathways. p < 0.05 was used as the cut-off criterion. After pathway enrichment analysis, we found that 160 DEGs were enriched in 11 pathways. Among them, DEGs were mainly enriched in the pathways in cancer (p = 0.03), transcriptional misregulation in cancer (p = 0.01), Rap1 signaling pathway (p = 0.02) and Ras signaling pathway (p = 0.03) (**Figure 3**).

### PPI Network Analysis

**Figure 4:**
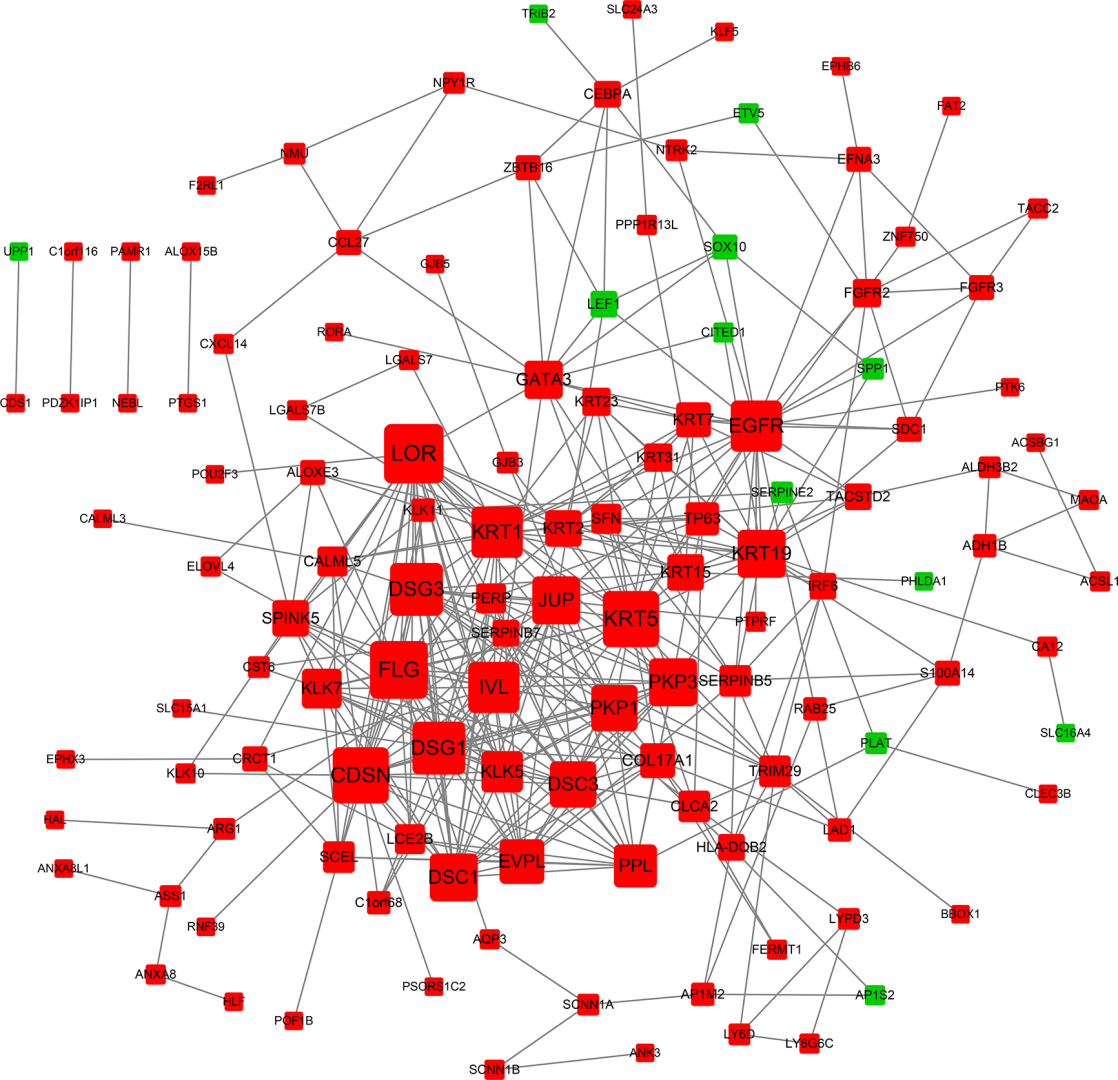
PPI network of DEGs. Each node is a differentially expressed gene (protein). A red node represents an up-regulated gene (protein). A green node represents a down-regulated gene (protein). The node size reflects node degree: the bigger the degree value, the bigger the node size is.

We obtained a PPI network from STRING to describe protein interactions (**Figure 4**). Based on the information obtained from the STRING database, a PPI framework with 160 nodes and 385 edges was generated, and its local clustering coefficient was 0.45. The results of computed hub genes were shown in the **Table 2**, including LOR, FLG, KRT5, CDSN, DSG1, DSG3, KRT1, IVL, and EGFR.

**Table 2:** The statistical results of connectivity degrees of the PPI network.

Gene	Degree
LOR	26
FLG	25
KRT5	24
CDSN	24
DSG1	22
DSG3	22
KRT1	21
IVL	21
EGFR	21

The gene in the table is the symbol of the protein (gene). Degree stands for the connectivity degree of the gene.

### Module Analysis of the PPI Network

**Figure 5:**
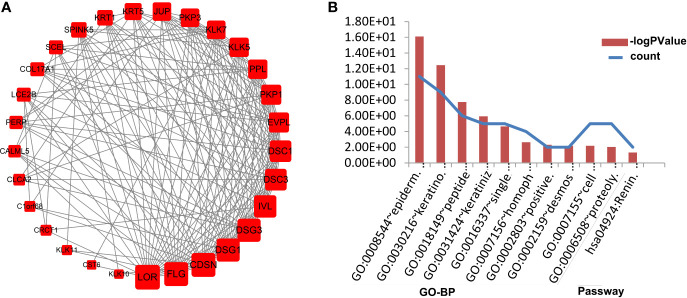
Functional enrichment analysis of the most important modules in PPI networks. The most significant module in the PPI network **(A)**. Nodes stand for the proteins (genes), and edges stand for the interactions of proteins. The GO analysis of the most significant module **(B)**. The left ordinate of histogram represents the gene counts, and the right represents the p-value. BP stands for biological process; and Pathway stands for cell signaling pathway.

To study and identify the function of the overlapping DEGs in detail, cluster analysis of the PPI network was conducted based on the ClusterONE Cytoscape plugin, an important tool for the analysis of densely connected and possibly overlapping regions within the Cytoscape network, which would contribute to the classification of protein network and relevant analysis. There were a total of 23 functional modules given and the most significant module (node = 29, density = 0.4335, p-value = 1.01E-8, **Figure 5A**) was selected for further analysis of functions and pathways to deeply understand the melanoma progression. To further verify the accuracy of this inference, the module genes were submitted into DAVID to perform the KEGG pathway enrichment analysis. The results showed that they were significantly enriched in the renin secretion signaling pathway, epidermal development, keratinocyte differentiation, peptide cross-linking, keratinization and single biological cell adhesion, among other processes shown, p < 0.05 (**Figure 5B**).

### Hub Genes Expression Level and Survival Analysis

**Figure 6:**
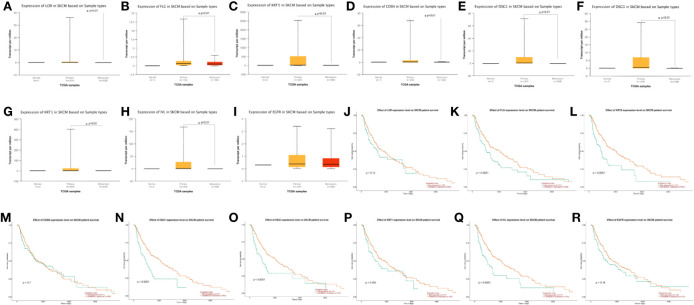
**(A-I)** Expression profile based on major sample types of hub genes using 473 patients data from TCGA database (*p < 0.01). **(J-R)** Kaplan-Meier survival plot of hub genes using 459 melanoma patients data from TCGA database (*p < 0.01).

Using UALCAN, we verified gene expression level of hub genes in 1 normal tissue, 104 primary tissues, and 368 metastasis tissues from TCGA database. Through this analysis, we found that LOR, FLG, KRT5, CDSN, DSG1, DSG3, KRT1, and IVL were closely related to the metastasis of melanoma (p < 0.01) (**Figures 6A–I**). Kaplan–Meier survival analyses showed that FLG, KRT5, DSG1, DSG3, and IVL expression levels were significantly associated with melanoma patient survival (p < 0.01) (**Figures 6J–R**).

### Detection of Hub Genes Related Protein Expression by Immunohistochemistry

**Figure 7:**
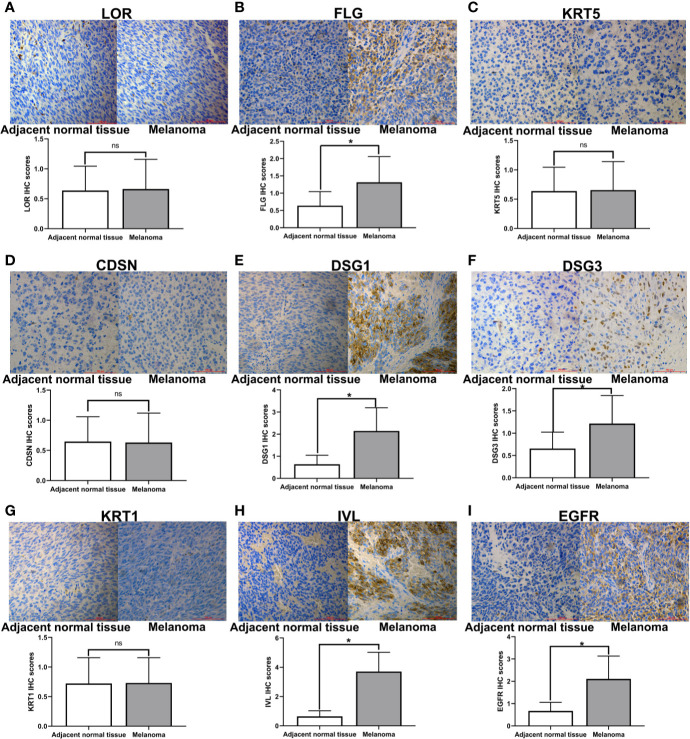
Immunohistochemical staining of hub genes in melanoma tissues and matched adjacent normal tissues. Representative examples of immunohistochemical assessment of hub genes (proteins) expression in melanoma tissues and matched adjacent normal tissues (magnification 200x), *p < 0.05 (ns, no significance).

In order to determine the expression of hub genes in human melanoma, we used immunohistochemical methods to detect the expression of the protein corresponding to hub genes. We detected the expression level of hub genes in 63 pairs of melanoma specimens (melanoma and adjacent normal tissue) by immunohistochemistry (**Figure 7**). The results showed that the expressions of FLG, DSG1, DSG3, IVL, and EGFR were considerably higher than those of adjacent normal tissues (P <0.05) (**Figures 7B, E, F, H, I**).

## Discussion

Melanoma is a progressive disease that requires effective prognostic indicators for diagnosis and treatment. In recent years, effective computational models have been constructed to identify disease-related mRNAs. However, most research has focused on using cell lines or animal models to intervene at the level of a single gene, protein, or miRNA (29, 30). Some commercial genetic testing kits for multi-center joint testing are still not comprehensive enough and still have deficiencies, and cannot effectively serve the purpose of detecting and discovering melanoma (31). For example, for clinicians currently using DecisionDx-melanoma, the integration of the results with the new AJCC staging standard is not clear, especially if the results of the 31 gene expression profile tests are inconsistent with the sentinel lymph node biopsy status (31). In our study, we used high-throughput expression data to study differential expression profiles of related genes in melanoma. We analyzed tumor and normal skin samples from patients in the GEO and TCGA databases to explore abnormally expressed genes in melanoma. The results showed that 160 differentially expressed genes were selected, including 144 up-regulated genes and 16 down-regulated genes. Later, we identified hub genes and pathways in melanoma based on the use of bioinformatics methods. We integrated 4 original microarray datasets and identified 160 frequently changed DEGs. DEGs were mainly enriched in 17 biological processes by GO terms, of which epidermis development, keratinocyte differentiation, keratinization, and establishment of skin barrier were the most obvious. KEGG pathway enrichment analysis showed that DEGs were mainly enriched in 5 signaling pathways, of which pathways in cancer, transcriptional misregulation in cancer, Rap1 signaling pathway and Ras signaling pathway were the most significant. In particular, the pathway of pathways in cancer was enriched by 8 DEGs, such as EGFR and JUP. EGFR (degree = 21) and JUP (degree = 19) were important key node genes in the PPI network. The results of our study suggested that these genes and pathways may play critical roles in the progression of melanoma. For instance, EGFR, as an essential receptor of transforming growth factor alpha, has attracted widespread attention. Previous studies have found that the frequency of oncogenic mutations in the EGFR gene is closely related to the occurrence of melanoma (32–34). In addition, EGFR has been proposed as an important molecular target for the treatment of cancer, which has promoted the development of EGFR pharmacological inhibitors (35, 36). Therefore, we speculated that EGFR may be a candidate gene in pathways in cancer of melanoma. Further, we used the TCGA database to detect the expression of hub genes. However, the sample size of non-melanoma normal tissues in the TCGA database is too small, with only one normal sample. When comparing tumor samples to normal samples, small sample sizes may cause inaccuracies, and we only compare primary and metastatic melanomas. The results showed that the expression of LOR, FLG, KRT5, CDSN, DSG1, DSG3, KRT1, and IVL in these nine hub genes were significantly different. In addition, we also explored the survival analysis of hub genes through the TCGA database. The results of the survival analysis showed that FLG, KRT5, DSG1, DSG3, and IVL of the nine central genes were notably related to the survival time of patients. Finally, based on the survival analysis, we performed clinical specimen validation using immunohistochemistry. The results showed that the expressions of FLG, DSG1, DSG3, IVL, and EGFR were markedly higher than those of adjacent normal tissues. These studies indicated that we got the key genes FLG, DSG1, DSG3, IVL, and EGFR that could affect melanoma development. We further discussed hub genes expressed in melanoma patient tissues. Filaggrin, a highly abundant protein of the stratum corneum, draw considerable attention after the discovery of its role in the aetiology of atopic dermatitis (37). Currently, Kezic S. has reported that FLG may serve as a potential biomarker for a reduced risk of melanoma (38), however, there are limited reports about the associations between FLG and melanoma. Thus, we can speculate that FLG may also play an important role in the melanoma progression as well as EGFR. Desmoglein belongs to the cadherin family, DSG1 and DSG3 are both members, and its intracellular part binds to intracellular anchoring proteins (39, 40). Li G et al. found that desmoglein and E-cadherin together act as an adhesion between factors, especially when melanoma cells proliferate, the expression of desmoglein and E-cadherin decreases (41). Das A et al. found that T-type calcium channel blockers inhibit autophagy and promote apoptosis of malignant melanoma cells (42). Therefore, calcium signaling pathway may be a disease-targeting in melanoma clinical trials. IVL is a soluble cytosolic protein with a molecular weight of 68KD (43). It is a substrate for glutamine transferase of keratinocytes and plays a role in the formation of epidermal keratinizing envelope (43, 44). IVL is synthesized in the spinal cell layer and cross-linked with granulosa cells under the action of glutamine transferase to form an important structural support for the skin barrier (45). At present, IVL is mostly used as a marker protein to study skin keratinocyte differentiation (46, 47). However, there are few reports of the associations between integrin and melanoma (48, 49). In view of its important role in maintaining skin function, we can speculate that IVL may also play an important role in the progression of melanoma. Although the research on key genes of melanoma has been studied, they are not comprehensive enough and lack of experimental verification (50, 51). In this study, a combined analysis of biology and experiment was used in order to have a more comprehensive and in-depth understanding of melanoma.

These limitations should be recognized by the current research. Firstly, in this study, only 160 DEGs were included. The prognostic Hub genes identified here may not represent all DEGs candidates that were potentially correlated with melanoma overall survival. Furthermore, this consideration does not include the location and stage of melanoma, because the information was not available for a considerable proportion of cases. At the same time, the functions of these eight Hub genes were inferred by bioinformatics analysis, and their biological roles in the development of melanoma were still unclear and should be explored in further experimental demonstration.

## Conclusion

In summary, we identified a signature of eight hub genes, which predicted the overall survival in four independent testing sets. Moreover, these hub genes were involved in cancer pathways, transcriptional dysregulation signaling pathways in cancer, Rap1 signaling pathway and Ras signaling pathway. These hub genes might well have clinical significance as diagnostic markers. This research was the first analysis of differential genes in melanoma and matched normal tissue samples. However, the biological roles of these eight hub genes in the occurrence and development of melanoma need further study.

## Data Availability Statement

Publicly available datasets were analyzed in this study. This data can be found here: https://www.ncbi.nlm.nih.gov/geo/, https://cancergenome.nih.gov/abouttcga/overview.

## Ethics Statement

The studies involving human participants were reviewed and approved by: The pathological experimental protocol was approved by the ethics committee of Kaifeng Central Hospital. The patients/participants provided their written informed consent to participate in this study.

## Author Contributions

YH and X-YJ conceived and designed this article. JY, CM, XW, HP, XZ, ZZ and BG participated in the experimental data collection. YH and XL coordinated all research realization steps, revised the study draft, and contributed to crucial discussion. All authors contributed to the article and approved the submitted version.

## Funding

This work was supported by the National Natural Science Foundation of China (No. 81670088 and No. 81602708); and the Henan Provincial Science Foundation Program (No.172102410019).

## Conflict of Interest

The authors declare that the research was conducted in the absence of any commercial or financial relationships that could be construed as a potential conflict of interest.
